# Supplementary figures and images for: A harmful religio-cultural practice (Chhaupadi) during menstruation among adolescent girls in Nepal: Prevalence and policies for eradication
Source: PLoS One. 2021 Sep 1;16(9):e0256968. doi: 10.1371/journal.pone.0256968 (PMC8409632; doi:10.1371/journal.pone.0256968)

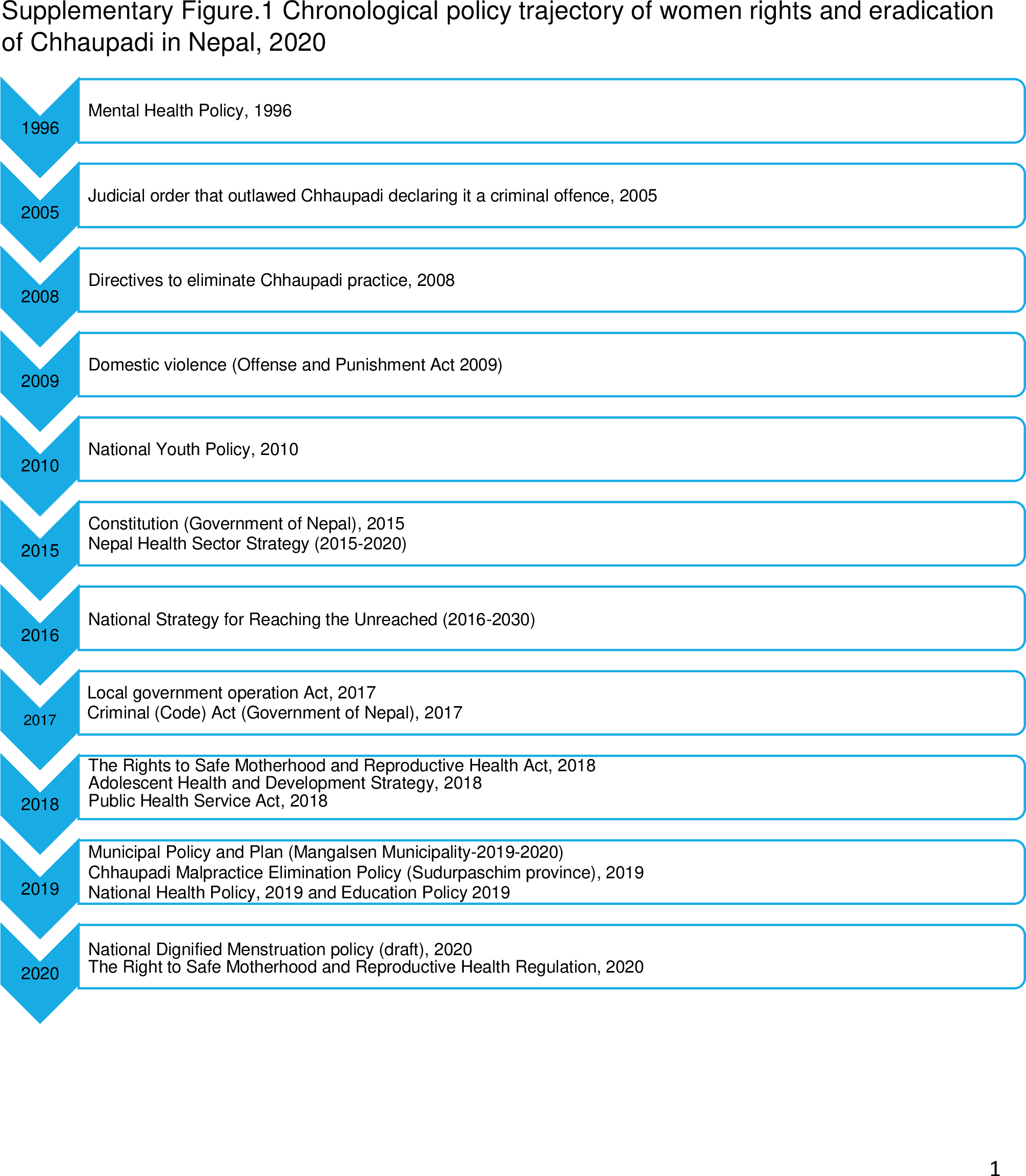

Supplement: S1 Fig — (TIF) [file pone.0256968.s001.tif]
