# Supplementary material for: A harmful religio-cultural practice (Chhaupadi) during menstruation among adolescent girls in Nepal: Prevalence and policies for eradication
Source: PLoS One. 2021 Sep 1;16(9):e0256968. doi: 10.1371/journal.pone.0256968 (PMC8409632; doi:10.1371/journal.pone.0256968)
Supplement: Attachment — Submitted filename: Reviewer Comments_April_22_2021.docx [file pone.0256968.s002.docx]

Thank you for the opportunity to review this timely and important piece of research. The study used a mixed methods approach to determine the magnitude of chhaupadi in a particular geographical area of Nepal and studied the policy context of the practice in the country. While there are many strengths of the study, there are several questions remaining and areas in which the manuscript could be improved for clarity. Below I outline some ideas for improving the manuscript for maximum impact. Overall, the manuscript requires a major revision.

**Overall Reviewer Comments:**

**Major**

- The two key study components, 1) the quantitative survey and 2) the policy review, feel like two separate studies. If being presented in the same manuscript, the authors should consider building a stronger justification for why these two areas in particular were studied, how the results of the two components build on/support each other (mixed methods integration) and discuss linkages between the results from both components. As it reads now the two pieces feel separate.

**Minor**

- Authors may carefully consider word choice to appropriately describe chhaupadi. For example, P2 Line 33, authors state chhaupadi is a century-old practice, however it is widely thought that the practice is “centuries-old.”
- In framing the study, it is suggested to clarify in the title that the study focuses on adolescent girls (i.e., the study does not measure the magnitude of chhaupadi exactly, rather it investigates the prevalence among adolescent girls in one particular area of the country and this could be clearly stated as to situate the reader more appropriately).
- A close reading to ensure language for grammar and accuracy would be beneficial (e.g., P2 line 51 is it that a girl is “from a nuclear family” or rather, “lives in a nuclear family”? P4 line 70 there are grammatical errors, etc.)
- While the study appears to be sound, the language is unclear throughout, making it difficult to follow. I advise the authors work with a writing coach or copyeditor to improve the flow and readability of the text.

**Below are specific comments for each section of the manuscript.**

**Introduction**

- P4 Line 74/75 is not clear (grammar/language issues).
- There are differing discussions in the literature regarding the translation/definition of the root words of “chhaupadi.” See NFCC, Bist, Baumann regarding definitions of chhaupadi. Chhau refers to a “woman’s condition as untouchable” and padi refers to a state of “becoming or being.”
  - Bist BS. The effect of religious hazards in health among menstrual women : A case of far-west Nepal. *Korean J Public Heal*. 2014;9.
  - Nepal Fertility Care Center. *Assessment Study on Chhaupadi in Nepal: Towards a Harm Reduction Strategy*. Kathmandu, Nepal; 2015.
  - Baumann, S., Lhaki, P., Terry, M., Sommer, M., Cutlip, T., Merante, M., & Burke, J. (2021) Beyond the Menstrual Shed: Exploring Caste/Ethnic and Religious Complexities of Menstrual Practices in Far-West Nepal. Women’s Reproductive Health.
- Updated literature search regarding menstrual restrictions would be beneficial for the reader (i.e., P4 lines 81-81, Amatya (in reference list but not cited here), Baumann 2021 (not in reference list, see above))
- For prevalence data, it would be more appropriate to cite the direct source of a research study, rather than the UN field bulletin. For example, P4 line 110 about chhaupadi prevalence in Accham, the UNICEF MICS would be a more appropriate and rigorous source.
- Considering the focus of this research on a policy review, a more extensive discussion of the chhaupadi policy history would be beneficial in the introduction. For example, at the end of the introduction, the authors mention policy loopholes, but a stronger discussion and more detailed information about the policies would help the reader understand the limitations in the current policy frameworks. Contextual discussion around the current Criminal Code 2074 is needed.
- Definition of the Criminal Code is incomplete. Fines are also a component. Ensure that the code is defined accurately and in full in the introduction.
- The study statement at the end of the introduction is clear. Nice work.

**Methods**

- A justification statement about the need for and use of mixed methods would add strength to the methods section. Furthermore, a discussion on methods integration would add merit, as it is mentioned in the figure but not discussed in the text. See Fetters and Creswell’s work on mixed methods integration at various states in the research design (<https://www.ncbi.nlm.nih.gov/pmc/articles/PMC4097839/>)
- Justify focus on adolescent girls specifically (and why not women as well?). Background on adolescent health should also be added to the introduction to prime the reader on this decision.
- Justify the focus on Achham district, and justify the purposive selection of Mangalsen? Why not select randomly, as was done for the wards?
- How was the list of all households with adolescent girls compiled/accessed?
- For the question, “person forcing to stay in chhaupadi huts?” was “self” an option? If not, why not? Or “other”? The question does not appear to have an exhaustive list of response options (if this cannot be addressed, it should be noted as a limitation). The same comment goes for “factors for chhaupadi practice.”
- Variable, “types of work restriction” appears to include items that are not work related, like wearing new clothes and taking medicine. Please rectify.
- Variable, absorbents, new clothes is mentioned twice.
- Were participants compensated in any way for their participation?
- Modifications regarding local dialect are mentioned, but then authors state the interviews were conducted in Nepali. Please clarify.
- Was the survey conducted on a tablet or pen and paper?
- Policy-cube framework requires a citation.
- Qualitative study documents should be listed. (P8 line 210).
- Figure 1 – Integration of quant and qual data- more detail required regarding **how** the data was integrated (see comment above about mixed methods integration and justifying this choice)
- Figure 2 – “Web searching” language is too vague to be helpful. It would be useful to know the search strategy, such as databases searched and keywords used, time the search was conducted.
- Authors state that the policy cube framework was modified for the study. How so?
  - Authors state they “coordinated with relevant stakeholders.” Do they mean “consulted”? Further, it would be helpful to know which stakeholders, and justify the choices made.
  - For “documents identified” add “Policy,” as the this document review was limited to policy documents, correct?
  - In the final selection box, it would be helpful to state the number that were included in the final review.

**Results**

- The authors state 84% practiced chhaupadi on their last menstruation, then state 94% practiced chhaupadi, which is confusing. Please clarify how the second question about practicing chhaupadi was asked.
- Dalit and Janajati are distinct ethnic groups with quite different menstrual practices in much of Nepal, based on what is known in the literature. Thus, it was surprising to see them combined for the quantitative analysis. Please justify this choice and discuss limitations of doing so.
- The way the results are discussed is confusing and contradictory at times, the results section should be revised for clarity in presentation of findings. For example, P11 line 275 states 84.8% practice chhaupadi, but at the end of the paragraph on line 281 the authors state 88.8% practice chhaupadi. This is either an error or the variables are not defined clearly in the text. Please rectify.
- Table 2
  - “Factors associated with chhaupadi” – Can the authors be more specific on what this is measuring? Are these reasons for why it is practiced? Clarity is necessary.
    - If this is asking the question, “Why do you practice chhaupadi?” 4 of the response options seem plausible but the response “lack of education” seems more like a demographic variable that could be compared with chhaupadi practice, rather than a response to this question in the survey. Did respondents say they practice because they are uneducated?
  - Some of the variables are non-exhaustive.
  - “Bathing in public sources of” appears to be incomplete.
  - “Work related restriction – response options do not all appear to be about “work.”
- Figure 3
  - Readability/formatting can be improved.
  - Full information about each policy/act is required. For example, 1996 simply states “Mental Health Policy,” which is not enough detail for the reader to find and review the document. Similarly, criminal code for 2017, the full details should be provided so reader is able to find more information as needed.
- General comment is that there are many tables and figures, some with overlapping information. Consider reducing/combining where possible. For example, Table 3 and figure 3 could likely be combined, as there is overlapping information.
- Formatting of table 3 could be improved (capitalization, adjusting width of columns, etc. to improve readability)
- Consistently provide both Nepali year and Gregorian year throughout manuscript.
- P14 lines 317-319 seems better suited for the introduction section, not the results.
- Criminal code details P15 lines 355-358- should specify 3 months imprisonment **and/or** a financial penalty

**Discussion**

- Prevalence – Authors state that other studies found lower prevalence rates. It would be helpful if the authors provided some discussion on why it may be the case that their particular study found such a high prevalence of the practice compared to other studies. Is there something about this particular area or period in which the study was conducted that may be unique?
- The results indicate a higher proportion of Dalit and Janajati practicing chhaupadi- how does this relates to what is known in the literature, and why this may this be the case in Achham context?
- It is interesting that caste/ethnicity did not play a role in chhaupadi practices, as other studies have found caste to be a significant predictor of menstrual practices in Nepal (Baumann et al. 2019) and India (Khanna et al. 2005). It will be important for the authors to compare their findings against other studies in health and menstruation that found caste/ethnicity to be a key variable and discuss why their findings may differ.
- Nuclear vs joint family – This is an interesting finding and glad to see that the authors contrasted these findings against another study that found the opposite.
- Comprehensiveness – p20 – While the authors are correct in stating that there is no gold standard in the literature for addressing chhaupadi specifically, authors could consider reviewing/comparting policies to actions that have been taken to address other harmful practices in Nepal and globally. For example, see dowry, child marriage etc. in UNFPA study: <https://nepal.unfpa.org/en/publications/literature-review-harmful-practices-nepal>
- It is suggested that the authors refrain from value-judgment language, and rather frame the issue in terms of health and rights violations. (e.g., P21 line 515 “bad cultural practice” could be replaced with “harmful cultural practice”)
- The finding regarding policy documents lacking a public reporting mechanism and independent monitoring system is a critical one. It would be helpful if the authors could expand upon this in the discussion (P22 Line 547) to discuss other policies/approaches that have worked effectively (even in context outside of Nepal if necessary) in order to frame the issue with a recommended step moving forward to creating adequate reporting mechanisms. What would/could a strong reporting mechanism look like for chhaupadi, and what can we learn from what has been done to address similar policy challenges for other issues?

**Strengths/Limitations**

- Authors state they employed a pretested survey, however, it would add to the strength if they could include more information in the methods section discussing how the survey questions were developed. Were they adapted from an existing pre-tested tool? Were the survey questions pulled from literature on the topic?
- “Critical analysis and synthesis of policies” is not necessarily a strength. This is expected in a content review of policy. If there were multiple independent reviewers of the policies it could be argued that would be an added strength to increase rigor and validity, but that doesn’t seem to be the case in this study.

**Policy implementations**

- Mental health is discussed here, if so, it should be supported by the results of the study.

**References**

- 7 – Link doesn’t work.
- 8 – Reference is incomplete.
- 22 – Provide a link to the code if possible (even if it is in Nepali)
- 44 – Name is in all caps- not consistent with other references.
- 40 – Remove “forthcoming” as this is already published.
- 48 – Reference is incomplete.
