# Supplementary material for: A harmful religio-cultural practice (Chhaupadi) during menstruation among adolescent girls in Nepal: Prevalence and policies for eradication
Source: PLoS One. 2021 Sep 1;16(9):e0256968. doi: 10.1371/journal.pone.0256968 (PMC8409632; doi:10.1371/journal.pone.0256968)
Supplement: Attachment — Submitted filename: Response to Reviwers.doc [file pone.0256968.s004.doc]

Reviewer #1

1. I wish congratulations to authors for their hard work and completing this research article. I was happy to review the article. And in my opinion, authors have done pretty good job in addressing the reviews and corrected every issues. So, I don't have any other comment regarding the article. This article is in line with PLOS ONE's criteria and fulfilled it.

*Authors response: Thank you so much for this compliment.*

Reviewer #2: Thanks to the authors for their rigorous dedication to addressing all the comments. Just a couple pending notes for the authors:

1. In reading through the manuscript, I still found a number of places where the grammar could be improved.

*Author’s response: Thank you so much for your suggestions. The final revised manuscript is edited for the flow of the language and grammatical errors. We got language and copy edits support from one of the bilingual (Nepali and English) academic as well as an associate editor of BMC Public Health.*

2. Citation 51 (page 23) is noted in text but not reference list.

*Author’s response****:*** *Thank you so much for your comment, we have reviewed it and corrected in the revised manuscript.*

3. The following study supports the author's findings about family influence, but is not referred to in the discussion, add it would help to build author's argument (Beyond the Menstrual Shed: Exploring Caste/Ethnic and Religious Complexities of Menstrual Practices in Far-West Nepal - Sara E. Baumann, Pema Lhaki, Martha A. Terry, Marni Sommer,Trevor Cutlip, Monica Merante, and Jessica G. Burke)

*Author’s response****:*** *Thank you so much for your comment, we have cited the suggested paper in the revised manuscript.*

We would like to thank both the reviewers for their insightful comments and feedback. Thank you so much for inviting us for revision of this manuscript.

Dipendra Singh Thakuri on behalf of all co-authors
